# Analysis of the OX40/OX40L immunoregulatory axis combined with alternative immune checkpoint molecules in pancreatic ductal adenocarcinoma

**DOI:** 10.3389/fimmu.2022.942154

**Published:** 2022-07-22

**Authors:** Xianlong Chen, Heng Ma, Shengwei Mo, Yue Zhang, Zhaohui Lu, Shuangni Yu, Jie Chen

**Affiliations:** Department of Pathology, Peking Union Medical College Hospital, Chinese Academy of Medical Sciences and Peking Union Medical College, Beijing, China

**Keywords:** OX40, OX40L, PD-L1, PDAC, immune checkpoint, immunotherapy, multiplexed immunofluorescence

## Abstract

Immune checkpoint modulation has been a vital therapeutic option in many malignancies, and targeting of novel immune checkpoints, including OX40/OX40L costimulatory signaling, is being assessed in clinical trials. However, little is known about the role of OX40 and OX40L in pancreatic ductal adenocarcinoma (PDAC). Thus, we investigated the clinical significance of OX-40 and OX40L and their associations with alternative immune checkpoints, immune infiltrates, clinicopathological features, and clinical outcomes. We performed multiplexed immunofluorescence staining for OX40, OX40L, CD8, and CD68 using tissue microarrays from 255 patients. Immunohistochemistry data for PD-L1, B7-H3, B7-H4, CD3, and Foxp3 were analyzed. And the RNA sequencing data of OX40/OX40L in The Cancer Genome Atlas and International Cancer Genome Consortium databases were also evaluated. The positive rates for OX40 on tumor cells (TCs) and immune cells (ICs) were 8.6% and 10.2%, respectively, and the positive rates for OX40L on TCs, ICs, and macrophages were 20%, 40.4%, and 12.9%, respectively. OX40 was associated with favorable clinicopathological features. OX40+ on ICs, OX40L+ on TCs, or OX40L+ on macrophages, rather than the total gene and protein levels of OX40/OX40L, were associated with improved survival. OX40+ on ICs and OX40L+ on macrophages were independent factors of clinical outcomes. Moreover, we could more accurately stratify patients through the combination of OX40 on ICs and OX40L on TCs, and patients with OX40+ ICs and OX40L+CK+ showed the best outcome. And we demonstrated that patients with OX40-ICs and low CD8+ T cells infiltration had unfavorable survival. Intriguingly, OX40+ ICs or OX40L+ macrophages demonstrated superior survival in patients with PD-L1 negativity than in those with PD-L1 positivity. Furthermore, OX40+ ICs were correlated with negative B7-H4 on TCs, high densities of CD3 T cells, and high densities of Foxp3 T cells; OX40+ TCs and OX40L+ TCs were associated with low densities of Foxp3 T cells. We identified OX40 and OX40L as promising predictors for prognosis in PDAC.

## Introduction

Pancreatic ductal adenocarcinoma (PDAC) accounts for more than 90% of all pancreatic malignancies (1). PDAC is the fourth leading cause of cancer-related deaths in developed countries, with a 5-year survival rate of less than 8%, and it is expected to be the third leading cause of cancer-related death in 2021 (2, 3). Because of resistance to currently available therapies and late diagnosis, the survival of patients with PDAC have made little improvement (4). Thus, new treatment strategies are urgently needed to improve the lives and prognosis of patients with PDAC.

Inhibiting programmed cell death 1 (PD-1) or its ligand programmed death-ligand 1 (PD-L1) has achieved remarkable success in some malignancies (5). In the KEYNOTE-028, the median progression-free survival (PFS) of PDAC patients treated with pembrolizumab was 1.7 months across 20 cancer types, and the objective response rate in 24 PDAC patients who were treated with pembrolizumab was 0% (6). Wainberg et al. demonstrated an overall response rate of 18% in patients with advanced PDAC treated with anti-PD-1 plus gemcitabine, however, the clinical results of this study were not supported by KEYNOTE-028 data (7). According to the data from these clinical trials, many patients with this disease do not experience benefits from blockade of PD-1/PD-L1, suggesting the existence of other immunosuppressive mechanisms in the tumor immune microenvironment.

OX40, also known as TNFRSF4 or CD134, is a member of the tumor necrosis factor receptor superfamily (8). OX40/OX40L signaling has been identified as one of the most important costimulatory pairs (9–11). OX40 is expressed on activated CD4+ and CD8+ T cells, leading to enhanced proliferation, survival, activation, and differentiation of effector T cells (9–11). OX40 also causes the generation of memory T cells (12). Additionally, OX40 expressed on CD4+Foxp3+ regulatory T cells (Tregs) inhibits the immunosuppressive function of Tregs (13). OX40L is mainly expressed by activated antigen-presenting cells, generating stimulatory signals to antigen-specific T cells through interaction with OX40 (10, 14, 15). Administration of agonist antibodies targeting OX40 has shown promising antitumor effects in animal models. Moreover, several clinical trials targeting OX40 as monotherapy or in combination with vaccination, radiotherapy, and anti-PD-1 or CTLA-4 are currently ongoing in a variety of types of tumors (16–20). To date, the expression patterns and clinical significance of the OX40/OX40L costimulatory axis remain unclear.

Recent studies have revealed that several factors can affect the response to immunotherapy, including the status of mismatch repair (MMR) and tumor-infiltrating immune cells (ICs) (21–23). Microsatellite instability-high or MMR-deficient patients showed a high response to targeting of PD-1/PD-L1 across 12 different cancer types, and p53, a classic tumor suppressor, induces PD-L1 expression in melanoma cells by modulating Janus kinase-2 expression (21, 22). In contrast, accumulating evidence shows that non-T cell-inflamed tumors, which are characterized by a low density of CD8+ T cells and low levels of activation and/or exhaustion of CD8+ T cells, are unresponsive to anti-PD-1 therapy (23). However, the association of OX40/OX40L expression with immune infiltrates and molecular status remain unknown in PDAC.

Given that OX40/OX40L are less studied immune checkpoints and have yet to be fully explored, especially in PDAC, we investigated OX40 and OX40L expression and simultaneously detected the status of other biomarkers in the tumor immune microenvironment using multiplexed immunofluorescence (mIF), digital imaging techniques, immunohistochemistry, and next-generation sequencing (NGS) data in PDAC samples and analyzed their correlations with immune markers (CD3, CD8, Foxp3, PD-L1, B7-H3, and B7-H4), and clinicopathological features and outcomes.

## Materials and methods

### Patients and tumor samples

This retrospective cohort was comprised of 255 patients with PDAC. All patients underwent surgery for PDAC from 2009 to 2019 in the Peking Union Medical College Hospital (Beijing, China) and were consecutively included in our current retrospective study. All patients underwent standard surgical procedures, including classic pancreaticoduodenectomy, pylorus-preserving pancreaticoduodenectomy, distal pancreatectomy, or total pancreatectomy. Clinicopathological parameters were collected from medical records, and medical record reviews and telephone interviews were used to obtain information on survival and recurrence. The time between surgery and tumor progression or the last follow-up appointment was defined as PFS. Disease-specific survival (DSS) was calculated from the date of surgery to the time of patient death caused by PDAC or the last follow-up, which was October 10, 2020. Follow-up data were available for all patients in this study. This study was approved by the Institutional Review Board of Peking Union Medical College Hospital (S-K1593) and conformed to the ethical standards set forth in the Declaration of Helsinki. Informed consent was obtained from all patients.

Tissue cylinders with a diameter of 2.0 mm each were punched from selected hematoxylin and eosin-stained paraffin blocks using a Tissue Microarrayer (MiniCore, Mitogen, Hertford, UK) and then re-embedded into recipient tissue microarray (TMA) blocks. All tumor spots were punched from the tumor center.

### The cancer genome atlas and international cancer genome consortium databases

To analyze the prognostic significance of mRNA from the OX40-encoding gene *TNFRSF2* and OX40L-encoding gene *TNFSF2* in PDAC, data from 181 patients with PDAC and 120 patients with PDAC were obtained from TCGA (https://tcga-data.nci.nih.gov/tcga/) and ICGC databases (https://dcc.icgc.org/), respectively. The patients in each set were divided into two groups: high vs. low expression) according to the median of mRNA levels.

### mIF staining and multispectral imaging

mIF staining was performed on TMA sections based on the manufacturer’s protocol (Opal Multiplex Immunohistochemistry Assay Kit, Akoya Biosciences, Marlborough, MA, USA) and Wand et al. to visualize the expression of OX40, OX40L, CD8, CD68, and CK (24). Briefly, TMA sections were baked at 65°C for 2 h, then dewaxed with xylene and rehydrated with a graded series of ethanol solutions of ethanol. We performed the heat-induced antigen retrieval in AR6 antigen retrieval buffer (pH, 6.0) for the aforementioned antibodies. Next, we blocked the TMA sections with blocking buffer (Dako, X0909) for 10 minutes, and then sections were incubated with the primary antibody for 1 hour at 37°C. In the first round of staining, anti-CK monoclonal antibody was used to localize the PDAC cells, followed by incubating the sections with an anti-rabbit horseradish peroxidase (HRP)-conjugated secondary antibody (Akoya Biosciences) for 10 minutes. The signal was further amplified using the Opal fluorophore working solution containing Opal 690 tyramide signal amplification (TSA) reagent (Akoya Biosciences). Subsequently, citrate buffer (pH, 6.0) was used to remove the bound antibody. The same procedures were repeated sequentially until all targets of interest were detected. The following antibodies were used: anti-CD68/Opal 540, anti-CD8/Opal 620, anti-OX40L/Opal 650, and anti-OX40/Opal 520. After five sequential rounds of staining, 4′,6-diamidino-2-phenylindole (DAPI) were applied to counterstain the slides for 5 minutes at room temperature. At last, the slides were mounted with a hard set medium. Details of the primary antibodies used are listed in **Supplementary Table S1**.

As described by Wang et al., the Vectra Polaris system (Akoya Biosciences) was used to scan the multiplex-stained TMA sections (24). According to the mode of TMA, the entire field of view for every core in each TMA section was acquired at 100 × multispectral images. Each image cube was established by combining images captured at 20-nm wavelength intervals from 420 to 720 nm. Images of a single stained section for each marker and those of an unstained section were applied to extract the spectrum of each fluorophore and those of the tissue autofluorescence, respectively, and to create a spectral library, which was then applied to separate each multispectral image cube into its individual components. Based on the inForm software (Akoya Biosciences), tissue-segmentation was performed according to cytokeratin and DAPI staining; cell-segmentation and phenotyping of individual cells were performed according to individual markers and presence of DAPI using Inform software. Thus, PDAC cells were considered as CK+, CD68 positivity represented macrophages, and positive CD8 expression represented cytotoxic T cells.

### Evaluation for OX40, OX40L, and tumor-infiltrating ICs

All analysis was independently performed by two investigators (XL. C. and H. M.) who were blinded to the patients’ clinicopathological data. Four random fields of view in each section were randomly acquired at 200 × multispectral images for quantitative digital analysis. The mean counts of four fields were used for statistical analysis. Samples were identified as positive OX40 expression on tumor cells (TCs) (OX40+/CK+) when ≥1% of the cells show OX40 staining and CK staining, regardless of OX40 positivity in non-TCs. We identified positive OX40 expression on ICs when ≥1% of the cells display OX40 staining in the tumor microenvironment (CK negative) and staining for DAPI. We defined OX40L positivity on TCs (OX40L+/CK+) as ≥1% of the cells displaying staining for OX40L and staining for CK, regardless of OX40L positivity in non-TCs. Samples were identified OX40L+ on ICs (OX40L+ ICs) when ≥1% of the cells that stained for OX40L in the tumor microenvironment (CK negative) and DAPI staining. OX40L positivity on macrophages was scored when ≥1% of the cells display staining for OX40L and staining for CD68. All cut-off values were set using the X-tile (Yale University, USA).

### Immunohistochemistry

The following primary antibodies were used for immunohistochemistry: PD-L1, B7-H3, B7-H4, CD3, CD8, and Foxp3. An automated immunostainer (BOND-III; Leica Biosystems, Wetzlar, Germany) was used for immunohistochemical staining, following the manufacturer’s standard protocols. We identified PD-L1 positivity on TCs through membrane staining and when TPS score was ≥1% based on clinical practice and previous studies (25, 26). Samples were considered as positive for B7-H3 and B7-H4 on TCs when ≥5% of the TCs expressed this protein. The 5% cut-off point was set using X-tile and was the best value for prognosis discrimination based on preliminary analysis of our cohort.

### Statistical analysis

The correlations between OX40 and OX40L and the clinicopathological characteristics, including molecular characteristics, alternative immune checkpoints, and immune infiltrates, were assessed using the χ² test. Survival curves were plotted using the Kaplan‒Meier method and compared using the log-rank test. Univariate and multivariate analyses were performed using the Cox proportional hazards model, and hazard ratios with 95% confidence intervals (CIs) for progression and death were calculated. Statistical significance was set at P<0.05. Statistical analyses were two-sided and performed using the Statistical Package for the Social Sciences (SPSS) software (version 22.0; SPSS, Inc., Chicago, IL, USA). Statistical analyses of NGS data from the ICGC and TCGA databases were conducted using R version 3.5.0 (http://www.r-project.org).

## Results

### Expression of OX40 and OX40L and their associations with clinicopathological characteristics in PDAC

**Figure 1:**
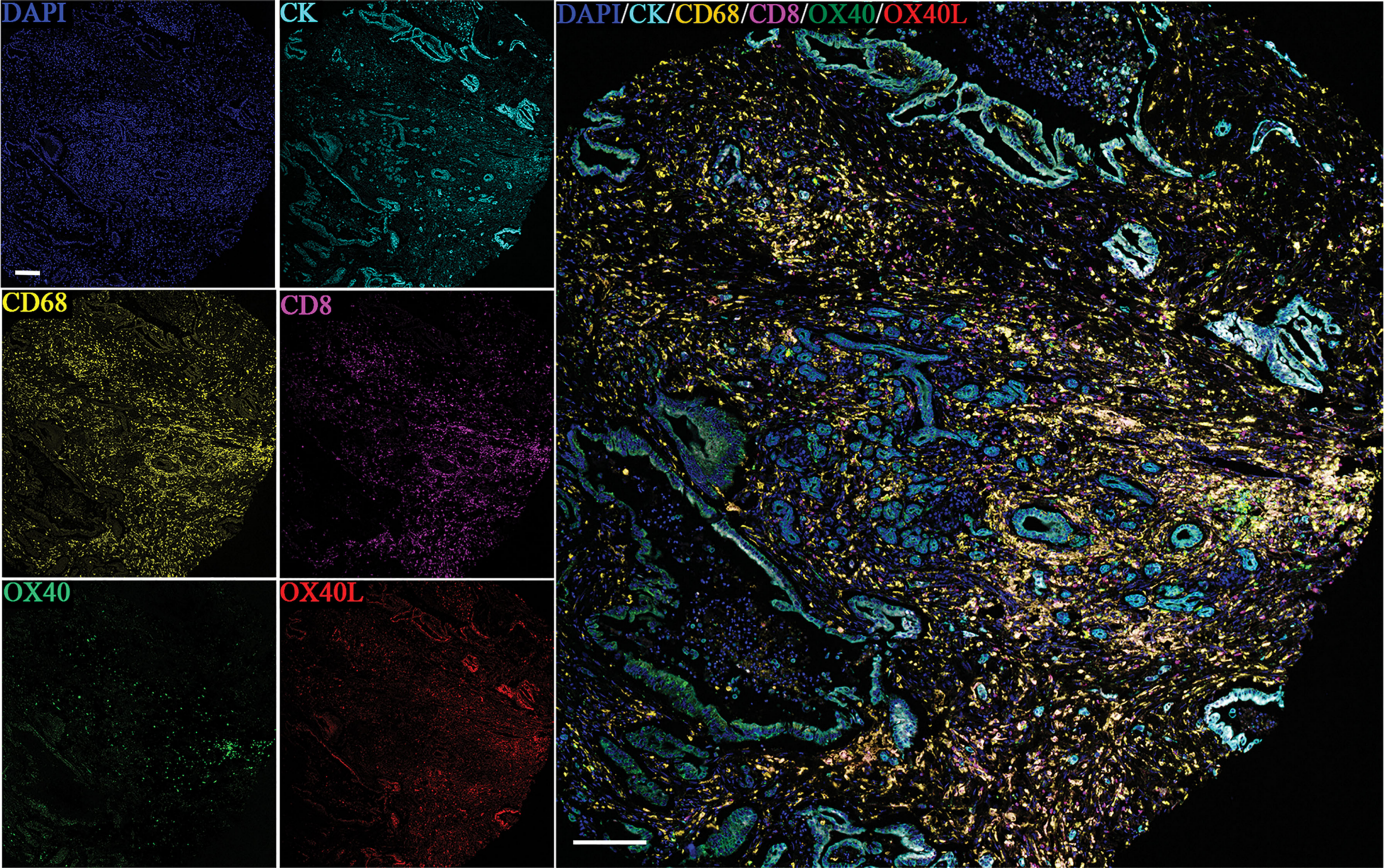
Six-color multispectral image and unmixed individual spectral images stained with 4′,6-diamidino-2-phenylindole (blue), CK (cyan), OX40 (green), OX40L (red), CD68 (yellow), and CD8 (purple). (200 × magnification).

**Figure 2:**
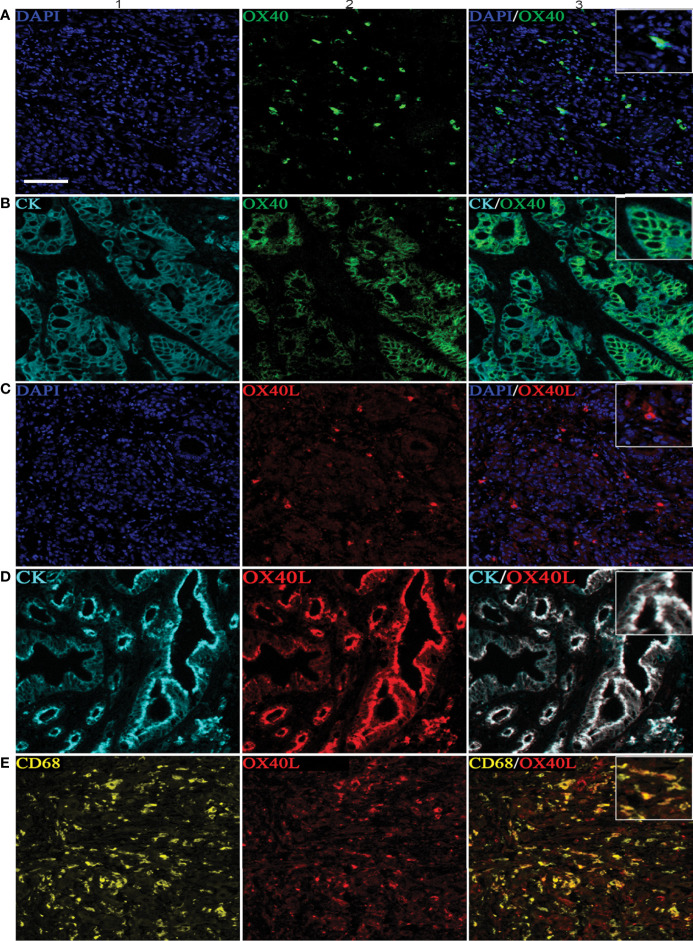
Positive OX40 expression on tumor cells (TCs) and immune cells (ICs) and positive expression of OX40L on TCs, ICs, and macrophages. Representative images of A1–A3, which are OX40 positive on ICs: individual component OX40 positivity on ICs obtained from multiplexed slides after spectral unmixing (OX40+, **A1**), cell nucleus (4′,6-diamidino-2-phenylindole [DAPI], **A2**), and two-color multispectral (OX40+ ICs, **A3**). Representative images of **(B1–B3)**, which are OX40 positive on TCs: OX40 positivity on TCs (OX40+, **B1**), tumor cell positivity (CK+, **B2**), and two-color multispectral imaging (OX40+/CK+, **B3**). Representative images of corresponding **(C1–C3)**, which are OX40L on ICs **(C1–C3)**. Representative images of corresponding **(D1–D3)**, which are OX40L on TCs **(D1–D3)**. Representative images of corresponding **(E1–E3)**, which are OX40L on macrophages **(E1–E3)**. DAPI (blue), CK (cyan), OX40 (green), OX40L (red), CD68 (yellow), and CD8 (purple). (200 × magnification).

Six-color multispectral images and separated individual spectral images stained with CK, DAPI, OX40, OX40L, CD8, and CD68 are shown in **Figure 1**. As shown in **Figures 2A, B**, OX40 was expressed in TCs and ICs. Positive OX40 on TCs and ICs was observed in 8.6% (22/255) and 10.2% (26/255) of PDAC samples, respectively. We also found positive OX40L expression in TCs and ICs; the OX40L positive rates on TCs and ICs were 20% (51/255) and 40.4% (103/255) (**Figures 2C, D**), respectively. Moreover, OX40L+ on macrophages was detected in 12.9% of cases (**Figure 2E**). Additionally, percentages of each component of the OX40+ cells and OX40L+ cells were shown in **Supplementary Figure S1**.

As shown in **Supplementary Tables S2** and **S3**, OX40+ on TCs was related to moderately/well-differentiated differentiation and more frequently observed in tumors with negative lymph node, OX40L positivity on TCs was associated with advanced tumor stage, and positive OX40L expression on both TCs and macrophages was more often observed in tumors located in the body or neck of the pancreas.

### Expression of OX40 and OX40L and survival

**Figure 3:**
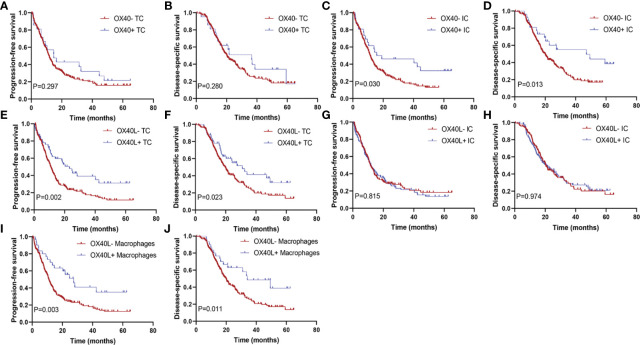
Kaplan–Meier curves according to OX40 and OX40L expressions. Progression-free survival for **(A)** OX40 expression on tumor cells (TCs), **(C)** OX40 expression on immune cells (ICs), **(E)** OX40L expression on TCs, **(G)** OX40L expression on ICs, and **(I)** OX40L expression on macrophages; disease-specific survival for **(B)** OX40 expression on TCs, **(D)** OX40 expression on ICs, **(F)** OX40L expression on TCs, **(H)** OX40L expression on ICs, and **(J)** OX40L expression on macrophages.

The median DSS of the cases with OX40+ ICs and those with OX40− ICs were 40.5 months (95% CI, 31.0–50.0) and 25.8 months (95% CI, 23.2–28.5), respectively. A similar result was observed for PFS. As shown in **Figures 3A–J**, positive OX40 on ICs was significantly correlated with favorable PFS and DSS; furthermore, patients with OX40L+ CK+ or OX40L+ CD68+ had significantly better PFS and DSS than those with OX40L− CK+ or OX40L− CD68+. However, we observed no significant difference in overall survival in 181 patients with PDAC from the TCGA cohort and 120 patients with PDAC from the ICGC cohort stratified by OX40 and OX40L gene expression levels based on the NGS data (**Supplementary Figures S2** and **S3**). Additionally, results of total OX40 or OX40L protein expression on prognosis were not significant in our internal queue data (unpublished data). Univariate analysis showed that a low differentiation grade, advanced American Joint Committee on Cancer (AJCC) stage, high T stage, high N stage, and distant metastasis were related to shorter PFS and DSS, whereas adjuvant chemotherapy OX40+ IC, OX40L+ TC, and OX40L+ macrophages were associated with improved PFS and DSS (**Table 1**). In multivariate analyses, OX40+ ICs or OX40L+macrophages were predictors of improved DSS independent of adjuvant chemotherapy, AJCC stage, or differentiation grade (**Table 2**).

**Table 1:** Univariate analysis of factors potentially associated with progression-free survival and disease-specific survival.

		Progression-free survival		Disease-specific survival	
Variables		HR (95% CI)	P-value	HR (95% CI)	P-value
Age, years	< 60 vs ≥ 60	1.13 (0.86–1.49)	0.378	1.17 (0.87–1.59)	0.303
Sex	Female vs male	1.42 (1.07–1.88)	**0.014**	1.34 (0.99–1.82)	0.056
Tumor differentiation	Moderately/well-differentiated vs poorly differentiated	1.77 (1.34–2.34)	< **0.001**	2.04 (1.51–2.75)	< **0.001**
Lymphovascular invasion	Absent vs present	1.24 (0.94–1.65)	0.127	1.21 (0.90–1.65)	0.214
Perineural invasion	Absent vs present	1.15 (0.86–1.55)	0.351	1.15 (0.83–1.58)	0.409
Tumor stage	T1-2 vs T3	1.60 (1.18–2.17)	**0.002**	1.75 (1.27–2.42)	**0.001**
Node stage	N0 vs N1-2	1.69 (1.26–2.26)	**< 0.001**	1.76 (1.27–2.44)	**0.001**
Distant metastasis	M0 vs M1	2.72 (1.48–5.03)	**< 0.001**	2.97 (1.61–5.51)	**< 0.001**
AJCC stage	I-II vs III-IV	2.02 (1.47–2.79)	**< 0.001**	2.13 (1.52–2.99)	**< 0.001**
Adjuvant chemotherapy	No vs yes	0.61 (0.50–0.82)	**< 0.001**	0.51 (0.37–0.69)	**< 0.001**
OX40 on TCs	Negative vs positive	0.76 (0.45–1.28)	0.299	0.74 (0.42–1.28)	0.282
OX40 on ICs	Negative vs positive	0.58 (0.35–0.96)	**0.033**	0.49 (0.28–0.87)	**0.015**
OX40L on TCs	Negative vs positive	0.55 (0.36–0.81)	**0.003**	0.62 (0.41–0.94)	**0.024**
OX40L on ICs	Negative vs positive	1.04 (0.77–1.39)	0.815	0.10 (0.72–1.39)	0.974
OX40L on Macrophages	Negative vs positive	0.47 (0.29–0.78)	**0.003**	0.51 (0.30–0.87)	**0.013**

AJCC, American Joint Committee on Cancer; CI, confidence interval; HR, hazard ratio; ICs, immune cells; TCs, tumor cells. P values <0 .05 are bolded.

**Table 2:** Multivariate analysis of factors potentially associated with progression-free survival and disease-specific survival.

	Progression-free survival		Disease-specific survival	
	HR (95% CI)	P-value	HR (95% CI)	P-value
Tumor differentiation		**0.001**		**< 0.001**
Moderately/well-differentiated	1		1	
Poorly differentiated	1.69 (1.25–2.28)		1.82 (1.31–2.51)	
AJCC stage		**0.005**		**0.006**
I-II	1		1	
III-IV	1.67 (1.17–2.39)		1.70 (1.17–2.47)	
Adjuvant chemotherapy		**0.012**		**0.002**
No	1		1	
Yes	0.67 (0.49–0.91)		0.58 (0.42–0.82)	
OX40 on ICs		0.085		**0.031**
Negative	1		1	
Positive	0.64 (0.38–1.06)		0.53 (0.29–0.94)	
OX40L on TCs		0.091		0.354
Negative	1		1	
Positive	0.69 (0.45–1.06)		0.80 (0.51–1.28)	
OX40L on Macrophages		0.328		**0.048**
Negative	1		1	
Positive	0.70 (0.41–1.21)		0.74 (0.41–1.33)	

AJCC, American Joint Committee on Cancer; CI, confidence interval; HR, hazard ratio; IC, immune cell; TCs, tumor cells. P values <0 .05 are bolded.

In subgroup analysis, tumors with OX40+ ICs or OX40L+TCs demonstrated superior PFS and DSS in early tumor stage (T1–T2) compared to those with OX40− ICs or OX40L−TCs, respectively. This prognostic benefit was not observed in late tumor stages (**Supplementary Figure S4**). However, tumors with OX40L+macrophages showed a more favorable prognosis in the late tumor stage (T3) than those with OX40L-negative macrophages; in the T1–T2 stage, there was no significant difference in PFS and DSS (**Supplementary Figure S4**).

### Co-expression of OX40L/CK on TCs and OX40 on ICs characterizes better outcome in PDAC

**Figure 4:**
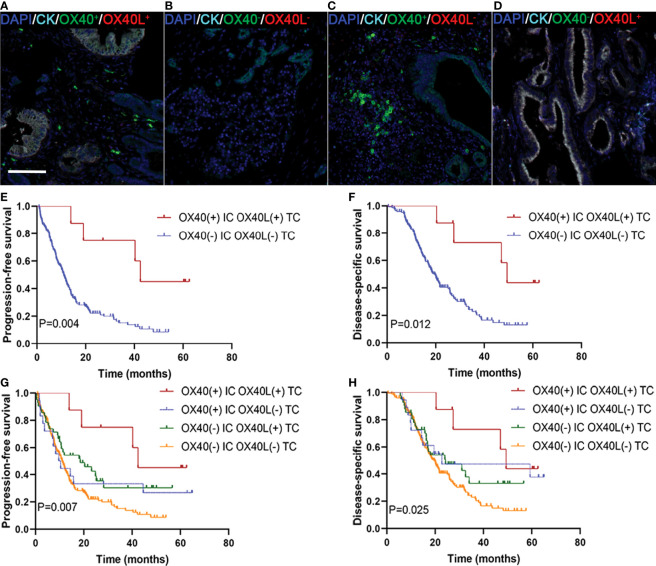
Localization of OX40 on immune cells (ICs) and OX40L on tumor cells (TCs) and their correlations with prognosis in PDAC. **(A)** OX40+ on ICs/OX40L+ on TCs, **(B)** OX40− on ICs/OX40L− on TCs, **(C)** OX40+ on ICs/OX40L− on TCs, and **(D)** OX40− on ICs/OX40L+ on TCs. Kaplan–Meier curves of PFS and DSS in patients with PDAC based on the combination of OX40 on ICs and OX40L on TCs **(E–H)**.

We explored whether the co-expression of OX40L on TCs and OX40 on ICs identified subgroups of patients with distinct clinical outcomes to further validate the effect of the OX40L/OX40 immunoactivation axis in PDAC. Among all 255 patients, 7 displayed OX40L+/CK+ as well as OX40+ ICs (**Figure 4A**), 185 exhibited OX40L−TC as well as OX40− ICs (**Figure 4B**), 19 exhibited OX40L−TC as well as OX40+ ICs (**Figure 4C**), and 44 displayed OX40L+TC and OX40− ICs (**Figure 4D**). Patients with OX40L+TC and OX40+ ICs had better PFS and DSS than those with OX40L−/CK+ and OX40− ICs (**Figures 4E, F**). Among these four groups, a significant difference in prognosis was observed, and we found that patients with OX40L−/CK+ and OX40− ICs showed the worst clinical outcomes, with a 3-year PFS rate of 13.6% and a 3-year DSS rate of 21.4% (**Figures 4G, H**).

### OX40 positivity on ICs, tumor-infiltrating CD8 T cells, and prognosis

**Figure 5:**
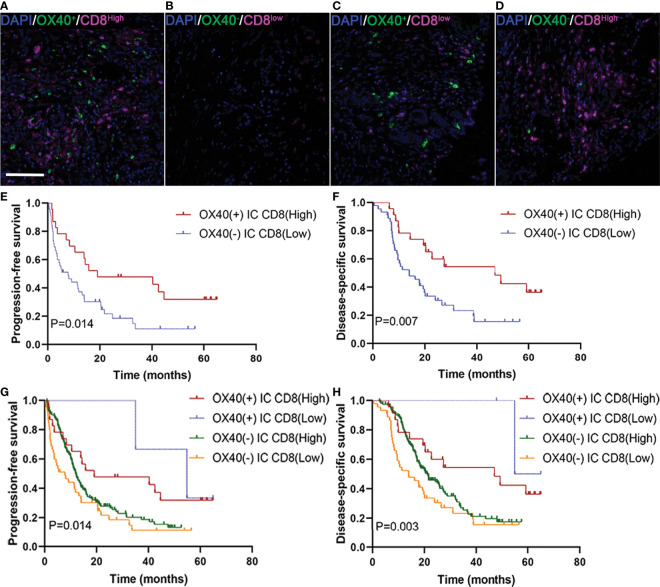
Localization of OX40 on immune cells (ICs) and CD8+ T cells and their correlations with prognosis in pancreatic ductal adenocarcinoma (PDAC). **(A)** OX40+ on ICs/CD8+ T cells^high^, **(B)** OX40+ on ICs/CD8+ T cells^low^, **(C)** OX40+ on ICs/CD8+ T cells^low^, and **(D)** OX40+ on ICs/CD8+ T cells^high^. Kaplan–Meier curves of progression-free survival and disease-specific survival in patients with PDAC based on the combination of OX40 on ICs and CD8+ T cells **(E–H)**.

We next studied whether the clinical significance of tumor-infiltrating CD8+ T cells affected the outcomes of patients with PDAC with OX40 positivity on ICs. In the present study, 24 revealed OX40+ ICs and CD8+ T cells ^high^ (**Figure 5A**), 47 displayed OX40− ICs and CD8+ T cells ^low^ (**Figure 5B**), 2 showed OX40+ ICs and CD8+ T cells ^low^ (**Figure 5C**), and 166 exhibited OX40− ICs and CD8+ T cells ^high^ (**Figure 5D**). OX40 positivity on ICs enhanced the favorable prognosis for patients, related to the presence of CD8+ T cells. Cases with OX40− ICs and low infiltration of CD8+ T cells showed the worst survival, whereas cases with OX40+ ICs and CD8+ T ^high^ cells showed the most favorable survival (**Figures 5E–H**).

### Correlations between OX40/OX40L and other immune markers of the tumor microenvironment

We investigated the associations of OX40/OX40L expression with other immune checkpoint molecules (PD-L1, B7-H3, and B7-H4). In the PDAC cohort, positive expression of PD-L1 and B7-H4 were correlated with poor PFS and DSS, whereas B7-H3 was associated with favorable PFS and DSS (these data currently under review). We found that OX40+ ICs were correlated with negative B7-H4 on TCs, high densities of CD3 T cells, and high densities of Foxp3 T cells; OX40+ TCs and OX40L+ TCs were associated with low densities of Foxp3 T cells (**Supplementary Tables S4** and **S5**). Moreover, in patients with negative PD-L1 expression, OX40+ IC or OX40L+ macrophages were significantly associated with prognosis; however, OX40+ IC or OX40L+ macrophages were not associated with clinical outcome in patients with positive PD-L1 expression (**Supplementary Figure S5**).

## Discussion

Targeting the OX40/OX40L signaling axis has been focused on for its clinical potential in improving the tumor immunosuppressive microenvironment (17). However, to date, we have known little about the clinicopathological significance of OX40 and OX40L in solid tumors, especially in PDAC. Herein, in a relatively larger cohort of 255 patients with PDAC, we revealed the clinical significance of the OX40/OX40L immune activation axis in PDAC. We found favorable survival for tumors with OX40+ on ICs, OX40L+ on TCs, or OX40L+ on macrophages. OX40+ ICs or OX40L+ TCs demonstrated superior survival in tumors with pT1–pT2 stage; in contrast, OX40L+ macrophages showed superior survival in tumors with pT3 stage. OX40+ ICs were correlated with negative B7-H4 on TCs, high densities of CD3 T cells, and high densities of Foxp3 T cells; OX40+ TCs and OX40L+ TCs were associated with low densities of Foxp3 T cells. Furthermore, we did not find any associations of OX40 and OX40L with other immune markers, such as PD-L1, B7-H3, and B7-H4.

The expression of OX40 and OX40L has been investigated in several other types of cancers (27–37). In our study, pathologists can easily identify the expression of OX40 and OX40L on ICs in PDAC only by evaluating OX40 or OX40L staining and cell morphology. However, we need more steps to identify the positive expression of OX40 and OX40L on TCs, given that it is validated through staining for OX40 or OX40L in combination with CK co-staining. Similar with OX40L expression on macrophages, it is also validated through staining for OX40L plus CD68 co-staining. In head and neck squamous cell carcinoma, acute lymphoblastic leukemia, and hepatocellular carcinoma, high OX40 expression is related to unfavorable clinical outcomes (27–29). However, high or positive OX-40 expression in tumor-infiltrating ICs is correlated with improved survival in cutaneous malignant melanoma, colorectal cancer, glioblastoma, non-small cell lung cancer, gastric cancer, and ovarian cancer (30–36). In our cohort, an improved prognosis was found in tumors with OX40 expression on ICs, and OX40+ TCs were not associated with DSS and PFS. However, OX40L was less studied compared to OX40. Shibahara et al. found that OX40L was associated with PFS in patients with glioblastoma (37), and OX40L expression on plasmacytoid dendritic cells promoted the progression of melanoma (38). We revealed that OX40L+ on TCs or macrophages was related to better PFS and DSS, whereas OX40L+ on ICs was not associated with survival in PDAC. Interestingly, according to NGS data from TCGA and ICGC databases, OX40 and OX40L gene expressions were not related to overall survival, and total OX40 or OX40L proteins were also not related to survival. These differences could be due to the following. First, NGS data are derived from bulk cells which result in limiting their analysis on the exact differentiation of TCs and ICs, similar with total proteins. OX40 and OX40L can be expressed on various types of cells, including TCs, ICs, and tumor-associated endothelial cells and tumor-associated microvessels (39), and the functions of OX40 or OX40L expressed on endothelial cells are independent of the costimulatory function of OX40/OX40L, which may not be associated with OX40/OX40L-mediated immunoactivities in PDAC. This affects the prognosis of patients. Second, the tumor microenvironment was complexed and variable due to spatial and temporal heterogeneities (40), and the number and distribution of OX40+ or OX40+ cells in the peripheral areas, tumor stroma, and tumor center, and may result in different growth patterns, which affect patients’ survival. These data demonstrated that it should precisely assess OX40L or OX40 expression on TCs or ICs, but not the total OX40/OX40L protein level or mRNA expression level in PDAC, specific to OX40/OX40L treatment in future clinical trials. Furthermore, we found that positive expression of OX40 on TCs was related to favorable clinicopathological features, including moderately/well-differentiated differentiation and negative lymph nodes, and it is different from the data for PD-L1 and B7-H4 in PDAC, PD-L1 and B7-H4 on TCs was associated unfavorable clinicopathological variables.

The OX40/OX40L signaling axis has been shown to perform its costimulatory functions to enhance the cytotoxic capacity of CD8+ T cells and generate memory T cells, and immunotherapy with OX40 agonists is becoming a new therapeutic strategy (39). Several OX40 agonists have been tested in a variety of types of malignant tumors (NCT01862900, NCT01303705, NCT02274155, NCT01644968, NCT02221960, NCT02318394, NCT02205333, and NCT02219724). Interestingly, our findings also demonstrated that OX40L positivity on TCs was positively correlated with OX40 positivity on ICs, and the administration of OX40 on ICs in combination with OX40L on TCs could identify 4 groups of PDAC patients with distinct prognoses. This finding suggests that the combined activation of OX40 and OX40L may be more potent than activation of either alone.

Several researches in the past few years have demonstrated the important clinical value of the different infiltrating patterns of tumor-infiltrating ICs (41, 42). Therefore, the assessment of targeted OX40L/OX40 costimulatory signaling treatments needs to combine the status of tumor-infiltrating ICs to identify patients with therapeutic potential for these strategies. In hepatocellular carcinoma and colorectal cancer, patients with positive expression of OX40 have higher densities of CD8 T cells (27, 32). In our study, OX40 expression on ICs was positively associated with densities of CD3+ T cells, and OX40+ ICs was also related to high densities of CD8+ T cells, although it was not significant; however, positive OX40 or OX40L on TCs was related to low densities of Foxp3 T cells, which may partly explain why OX40 and OX40L were associated with improved survival. Moreover, patients with PDAC with OX40+ ICs and high infiltration of CD8+ T cells showed the best outcome, in contrast, patients with OX40− on ICs and low infiltration of CD8 T cells showed the most unfavorable PFS and DSS. Immunoactivation of the OX40/OX40L axis may recruiting antitumor CD8 T-cell to tumors in PDAC, and increase the capacity of cytotoxicity and proliferation of CD8+ T cells. Thus, OX40 expression on ICs was stratified by CD8 T cells in our study, and we introduced an interaction term to assess the prognostic effect in CD8-low or CD8-high groups. On the basis of the aforementioned results, OX40 expression in ICs was modified by CD8 T cells. However, the regulatory mechanisms between OX40 and immune infiltrates remain unknown in PDAC, and more in-depth studies should be laid out to investigate this mechanism in the future. Furthermore, growing evidence revealed that spatial distribution and interactions between TCs and ICs, along with interactions among ICs, play key roles in cancer immunotherapy (43). Therefore, the spatial relationship between OX40L+ TCs and OX40+ ICs should further explore in a follow up paper to better understand clinical implications of spatial variation in PDAC.

The correlation between OX40 and other alternative immune checkpoints has been investigated in several types of cancer (27, 30). Xie et al. revealed that high expression of OX40 in the tumor microenvironment was associated with high densities of PD-1+ lymphocytes in hepatocellular carcinoma (27). Massarelli et al. observed an independent prognostic feature of OX40 when they assessed the effect of PD-L1 co-expression, suggesting that OX40 is a stronger driver of survival than PD-L1 in non-small-cell lung cancer (30). We believe that these results are of particular interest given the ongoing clinical trials of OX40 agonists plus PD-1 inhibitors. In our study, we found that expression of OX40 and OX40L on TCs or ICs was not associated with PD-L1 expression on TCs; interestingly, OX40+ on ICs or OX40L+ on macrophages demonstrated a superior survival in patients with PD-L1-negative PDAC, whereas no significant difference in survival was observed in patients with PD-L1-positive PDAC. Messenheimer et al. found that the concurrent addition of PD-1 inhibitors significantly improved the therapeutic effect of OX40 agonist alone in MMTV-PyMT tumors (44). Consistent with that study, Guo et al. also revealed that a combination of OX40 agonists and PD-1 inhibitors apparently increased survival in preclinical models of ovarian cancer (19). Moreover, in mice, combination therapy with OX40 agonist and PD-1 antagonist extends overall survival and eradicates PDAC through reducing the proportion of Tregs in PDAC and increasing numbers of memory CD4+ and CD8+ T cells (45). These data may support the combined targeting of PD-L1/PD-1 and OX40 as a therapeutic option. In addition, we found that OX40 positivity in the tumor microenvironment was associated with negative B7-H4 expression on TCs in PDAC. As known to us, B7-H4 is a T-cell coinhibitory B7 family molecule (46). However, the regulatory mechanisms between B7-H4 and OX40 remain unclear. Further studies are warranted to validate these findings.

PDAC is known for its desmoplastic stroma reaction composed of cancer-associated fibroblast, immune cells, and extracellular matrix, and cancer-associated fibroblasts, which are a major component of stroma in PDAC, have gained attention in recent years (47). Previous studies revealed a correlation between unfavorable clinical outcome and composition and quantity of fibroblast as well as immune cells, resulting in a weaker adaptive immune response in PDAC (47, 48). Li et al. showed that upregulation of OX40L in cancer-associated fibroblast facilitates chemoresistance of lung adenocarcinoma through inhibiting apoptosis of tumor cells (49). Elhai et al. blockade of OX40L is a promising strategy for the treatment of inflammation-driven fibrosis and OX40L was identified as a promising serum biomarker to predict the worsening of lung and skin fibrosis (50). These data highlight the role of OX40-OX40L pathway in fibrosis and/or fibroblast. Although, in this study, the expression of OX40L/OX40 in fibroblast were not explored in PDAC, the association of OX40L/OX40 with fibroblast and their regulatory mechanism should be investigated in the future.

Although there were some valuable findings in this study, some limitations still exist. First, it was a retrospective study. Second, tumor heterogeneity was inevitable owing to the use of TMAs. However, many studies detecting immune checkpoints in different tumors using TMAs have shown consistent results (27, 34). Finally, this study was performed in patients from a single institution and lacked an independent validation cohort. However, we used NGS data to analyze the prognostic value of OX40/OX40L in two independent cohorts from the TCGA and ICGC databases.

In conclusion, we found that both OX40 and OX40L were expressed on TCs and ICs, and OX40L was also detected in macrophages. OX40+ on ICs, OX40L+ on TCs, or OX40L+ on macrophages were associated with better DSS and PFS. Moreover, both OX40+ on ICs and OX40L+ on macrophages were independent factors for a favorable prognosis. Additionally, we found that four subgroups based on OX40 on ICs and OX40L on TCs (or CD8+ T cells) revealed distinct clinicopathological characteristics. Therefore, immunoactivation and immunosuppression are closely associated with PDAC, and targeting these two aspects may offer an effective therapeutic strategy against PDAC.

## Data availability statement

The original contributions presented in the study are included in the article/**Supplementary material**. Further inquiries can be directed to the corresponding author.

## Author contributions

XC and HM contributed to sample and data acquisition and manuscript drafting. SM, YZ, SY, and ZL offered technical support. JC made substantial contributions to the conception design of the study, funding the study, and supervision. All authors read and approved the final manuscript. All authors contributed to the article and approved the submitted version.

## Funding

This work was supported by grants from the Chinese Academy of Medical Sciences Initiative for Innovative Medicine (CAMS-2016-I2M-1–001), the National Natural Science Foundation of China (Nos. 81472326 and 81672648), and the National Scientific Data Sharing Platform for Population and Health (NCMI-YF01N-201906).

## Conflict of interest

The authors declare that the research was conducted in the absence of any commercial or financial relationships that could be construed as a potential conflict of interest.

## Publisher’s note

All claims expressed in this article are solely those of the authors and do not necessarily represent those of their affiliated organizations, or those of the publisher, the editors and the reviewers. Any product that may be evaluated in this article, or claim that may be made by its manufacturer, is not guaranteed or endorsed by the publisher.
