# Supplementary material for: Analysis of the OX40/OX40L immunoregulatory axis combined with alternative immune checkpoint molecules in pancreatic ductal adenocarcinoma
Source: Front Immunol. 2022 Jul 22;13:942154. doi: 10.3389/fimmu.2022.942154 (PMC9352865; doi:10.3389/fimmu.2022.942154)

| **Supplementary Table S1**. Details of antibodies used for multiplexed immunofluorescence and immunohistochemical staining | | | | |
| --- | --- | --- | --- | --- |
| **Protein** | **Clone** | **Dilution** | **Source** | **Catalog number** |
| OX40 | Rabbit monoclone [E9U7O] | 1:50 | Cell Signaling Technology | 61637 |
| OX40L | Rabbit monoclone [D6K7R] | 1:200 | Cell Signaling Technology | 59036 |
| CK | Mouse monoclone [C11] | 1:250 | Cell Signaling Technology | 4545 |
| PD-L1 | Rabbit monoclone [E1L3N] | 1:200 | Cell Signaling Technology | 13684 |
| B7-H3 | Rabbit monoclone [D9M2L] | 1:200 | Cell Signaling Technology | 58798 |
| B7-H4 | Rabbit monoclone [D1M8I] | 1200 | Cell Signaling Technology | 14572 |
| CD3 | Rabbit monoclone [SP7] | 1:100 | Abcam | ab16669 |
| CD8 | Rabbit monoclone [EPR21769] | 1:2000 | Abcam | ab217344 |
| CD68 | Mouse monoclone [KP1] | 1:3000 | Abcam | ab955 |
| Foxp3 | Mouse monoclone [236A/E7] | 1:500 | Abcam | ab20034 |
|  | |  |  |  |

**Supplementary Table S2.** Association between OX40 and clinicopathological features (n = 255)

| Variables | N | OX40 on TCs | | | OX40 on ICs | | |
| --- | --- | --- | --- | --- | --- | --- | --- |
|  |  | Negative | Positive | P value | Negative | Positive | P value |
| Sex |  |  |  | 0.372 |  |  | 0.626 |
| Female | 116 | 104(90) | 12(10) |  | 103(89) | 13(11) |  |
| Male | 239 | 129(96) | 10(4) |  | 126(95) | 13(5) |  |
| Age, years |  |  |  | 0.448 |  |  | 0.679 |
| <60 | 108 | 97(90) | 11(10) |  | 96(89) | 12(11) |  |
| ≧60 | 147 | 136(93) | 11(7) |  | 133(90) | 14(10) |  |
| Location |  |  |  | 0.504 |  |  | 0.189 |
| Head | 156 | 144(92) | 12(8) |  | 137(88) | 19(12) |  |
| Body&neck | 99 | 89(90) | 10(10) |  | 92(93) | 7(7) |  |
| Lymphovascular invasion | |  |  | 0.405 |  |  | 0.156 |
| Absent | 160 | 148(93) | 12(7) |  | 147(92) | 13(8) |  |
| Present | 95 | 85(89) | 10(11) |  | 82(86) | 13(14) |  |
| Perineural invasion |  |  |  | 0.241 |  |  | 0.955 |
| Absent | 87 | 77(89) | 10(11) |  | 78(90) | 9(10) |  |
| Present | 168 | 156(93) | 12(7) |  | 151(90) | 17(10) |  |
| Tumor differentiation |  |  |  | **0.017** |  |  | 0.893 |
| Moderately/well-differentiated | 160 | 141(88) | 19(12) |  | 144(90) | 16(10) |  |
| Poorly differentiated | 95 | 92(97) | 3(3) |  | 85(89) | 10(11) |  |
| Tumor stage | |  |  | 0.876 |  |  | 0.078 |
| T1-2 | 189 | 173(92) | 16(8) |  | 166(88) | 23(12) |  |
| T3 | 66 | 60(91) | 6(9) |  | 63(95) | 3(5) |  |
| Lymph node  metastasis |  |  |  | **0.041** |  |  | 0.642 |
| Absent | 99 | 86(87) | 13(13) |  | 90(91) | 9(9) |  |
| Present | 156 | 147(94) | 9(6) |  | 139(89) | 17(11) |  |
| Distant metastasis |  |  |  | 0.348 |  |  | 0.604 |
| M0 | 246 | 224(91) | 22(9) |  | 220(89) | 26(11) |  |
| M1 | 9 | 9(100) | 0(0) |  | 9(100) | 0(0) |  |
| AJCC stage |  |  |  | 0.860 |  |  | 0.638 |
| Ⅰ-Ⅱ | 205 | 187(91) | 18(9) |  | 185(90) | 20(10) |  |
| Ⅲ- Ⅳ | 50 | 46(92) | 4(8) |  | 44() | 6() |  |

AJCC, American Joint Committee on Cancer; TC, tumor cell; IC, immune cell.

**Supplementary Table S3.** Association between OX40L and clinicopathological parameters (n = 255)

| Variables | N | OX40L on TCs | | | OX40L on ICs | | | OX40L on Macrophages | | |
| --- | --- | --- | --- | --- | --- | --- | --- | --- | --- | --- |
|  |  | Negative | Positive | P value | Negative | Positive | P value | Negative | Positive | P value |
| Sex |  |  |  | 0.314 |  |  | 0.582 |  |  | 0.705 |
| Female | 116 | 96 (83) | 20(17) |  | 67(58) | 49(42) |  | 102(88) | 14(12) |  |
| Male | 139 | 108(78) | 31(22) |  | 85(61) | 54(39) |  | 120(86) | 19(14) |  |
| Age, years |  |  |  | 0.254 |  |  | 0.722 |  |  | 0.993 |
| <60 | 108 | 90(83) | 18(17) |  | 63(58) | 45(42) |  | 94(87) | 14(13) |  |
| ≧60 | 147 | 114(78) | 33(22) |  | 89(61) | 58(39) |  | 128(87) | 19(13) |  |
| Location |  |  |  | **0.008** |  |  | 0.998 |  |  | **0.047** |
| Head | 156 | 133(85) | 23(15) |  | 93(60) | 63(40) |  | 141(90) | 15(10) |  |
| Body&neck | 99 | 71(72) | 28(28) |  | 59(60) | 40(40) |  | 81(82) | 18(18) |  |
| Lymphovascular invasion |  |  |  | 0.517 |  |  | 0.922 |  |  | 0.910 |
| Absent | 160 | 126(79) | 34(21) |  | 95(59) | 65(41) |  | 139(87) | 21(13) |  |
| Present | 95 | 78(82) | 17(18) |  | 57(60) | 38(40) |  | 83(87) | 12(13) |  |
| Perineural invasion |  |  |  | 0.597 |  |  | 0.191 |  |  | 0.771 |
| Absent | 87 | 68(78) | 19(22) |  | 47(54) | 40(46) |  | 75(86) | 12(14) |  |
| Present | 168 | 136(81) | 32(19) |  | 105(62) | 63(38) |  | 147(87) | 21(13) |  |
| Tumor differentiation |  |  |  | 0.517 |  |  | 0.922 |  |  | 0.618 |
| Moderately/well-differentiated | 160 | 126(79) | 34(21) |  | 95(59) | 65(41) |  | 138(86) | 22(14) |  |
| Poorly differentiated | 95 | 78(82) | 17(18) |  | 57(60) | 38(40) |  | 84(88) | 11(12) |  |
| Tumor stage | |  |  | **0.038** |  |  | 0.330 |  |  | 0.534 |
| T1-2 | 189 | 157(83) | 32(17) |  | 116(61) | 73(39) |  | 166(88) | 23(12) |  |
| T3 | 66 | 47(71) | 19(29) |  | 36(55) | 30(45) |  | 56(85) | 10(15) |  |
| Lymph node  metastasis |  |  |  | 0.095 |  |  | 0.791 |  |  | 0.943 |
| Absent | 99 | 74(75) | 25(25) |  | 58(59) | 41(41) |  | 86(87) | 13(13) |  |
| Present | 156 | 130(83) | 26(17) |  | 94(60) | 62(40) |  | 136(87) | 20(13) |  |
| Distant metastasis |  |  |  | 0.865 |  |  | 0.491 |  |  | 0.610 |
| M0 | 246 | 197(80) | 49(20) |  | 148(60) | 98(40) |  | 213(87) | 33(13) |  |
| M1 | 9 | 7(78) | 2(22) |  | 4(44) | 5(56) |  | 9(100) | 0(0) |  |
| AJCC stage |  |  |  | 0.115 |  |  | 0.367 |  |  | 0.825 |
| Ⅰ-Ⅱ | 205 | 160(78) | 45(22) |  | 125(61) | 80(39) |  | 178(87) | 27(13) |  |
| Ⅲ- Ⅳ | 50 | 44(88) | 6(12) |  | 27(54) | 23(46) |  | 44(88) | 6(12) |  |

AJCC, American Joint Committee on Cancer; TC, tumor cell; IC, immune cell.

**Supplementary Table S4.** OX40, immune markers, TP53, and mismatch repair status in pancreatic ductal adenocarcinoma

| Variables | N | OX40 on TCs | | | OX40 on ICs | | |
| --- | --- | --- | --- | --- | --- | --- | --- |
|  |  | Negative | Positive | P value | Negative | Positive | P value |
| PD-L1 (N=235) | |  |  | 0.336 |  |  | 0.102 |
| Negative | 164 | 151(92) | 13(8) |  | 143(87) | 21(13) |  |
| Positive | 71 | 63(89) | 8(11) |  | 67(94) | 4(6) |  |
| B7-H3 (N=209) |  |  |  | 0.520 |  |  | 0.602 |
| Negative | 8 | 7(88) | 1(12) |  | 8(100) | 0(0) |  |
| Positive | 201 | 184(92) | 17(8) |  | 178(89) | 23(11) |  |
| B7-H4 (N=223) |  |  |  | 0.064 |  |  | **0.046** |
| Negative | 52 | 44(85) | 8(15) |  | 42(81) | 10(19) |  |
| Positive | 171 | 159(93) | 12(7) |  | 155(91) | 16(9) |  |
| CD3 (N=246) |  |  |  | 0.561 |  |  | **0.048** |
| Low | 57 | 53(93) | 4(7) |  | 55(96) | 2(4) |  |
| High | 189 | 171(90) | 18(10) |  | 165(87) | 24(13) |  |
| CD8 (N=239) |  |  |  | 0.460 |  |  | 0.087 |
| Low | 49 | 46(94) | 3(6) |  | 47(96) | 2(4) |  |
| High | 190 | 172(91) | 18(9) |  | 166(87) | 24(13) |  |
| Foxp3 (N=201) |  |  |  | **0.015** |  |  | **0.044** |
| Low | 97 | 84(87) | 13(13) |  | 92(95) | 5(5) |  |
| High | 104 | 100(96) | 4(4) |  | 90(87) | 14(13) |  |

PD-L1, programmed cell death-ligand 1; B7-H3, B7 homolog 3; B7-H4, B7 homolog 4; TC, tumorl cell; IC, immune cell.

**Supplementary Table S5.** OX40L, immune markers, TP53, and mismatch repair status in pancreatic ductal adenocarcinoma

| Variables | N | OX40L on TCs | | | OX40L on ICs | | | OX40L on Macrophages | | |
| --- | --- | --- | --- | --- | --- | --- | --- | --- | --- | --- |
|  |  | Negative | Positive | P value | Negative | Positive | P value | Negative | Positive | P value |
| PD-L1 (N=235) |  |  |  | 0.860 |  |  | 0.685 |  |  | 0.742 |
| Negative | 164 | 130(79) | 34(21) |  | 100(61) | 64(39) |  | 143(87) | 21(13) |  |
| Positive | 71 | 57(80) | 14(20) |  | 39(55) | 32(45) |  | 63(89) | 8(11) |  |
| B7-H3 (N=209) |  |  |  | 0.262 |  |  | 0.788 |  |  | 0.600 |
| Negative | 8 | 5(63) | 3(37) |  | 5(63) | 3(37) |  | 8(100) | 0(0) |  |
| Positive | 201 | 159(79) | 42(21) |  | 116(58) | 85(42) |  | 176(88) | 25(12) |  |
| B7-H4 (N=223) |  |  |  | 0.398 |  |  | 0.521 |  |  | 0.724 |
| Negative | 52 | 43(83) | 9(17) |  | 33(63) | 19(37) |  | 44(85) | 8(15) |  |
| Positive | 171 | 132(77) | 39(23) |  | 100(58) | 71(42) |  | 148(87) | 23(13) |  |
| CD3 (N=246) |  |  |  | 0.894 |  |  | 0.675 |  |  | 0.548 |
| Low | 57 | 46(81) | 11(19) |  | 32(56) | 25(44) |  | 48(84) | 9(16) |  |
| High | 189 | 151(80) | 38(20) |  | 112(59) | 77(41) |  | 165(87) | 24(13) |  |
| CD8 (N=239) |  |  |  | 0.279 |  |  | 0.455 |  |  | 0.865 |
| Low | 49 | 36(73) | 13(27) |  | 31(63) | 18(37) |  | 43(88) | 6(12) |  |
| High | 190 | 153(81) | 37(19) |  | 109(57) | 81(43) |  | 165(85) | 25(15) |  |
| Foxp3 (N=201) | |  |  | **0.035** |  |  | 0.250 |  |  | 0.818 |
| Low | 97 | 68(70) | 29(30) |  | 50(52) | 47(48) |  | 85(88) | 12(12) |  |
| High | 104 | 86(83) | 18(17) |  | 62(60) | 42(40) |  | 90(87) | 14(13) |  |

PD-L1, programmed cell death-ligand 1; B7-H3, B7 homolog 3; B7-H4, B7 homolog 4; TC, tumor cell; IC, immune cell.

**Supplementary Figure S1**. Percentage of each component of the (A) OX40+ cells and (B) OX40L+ cells.

**
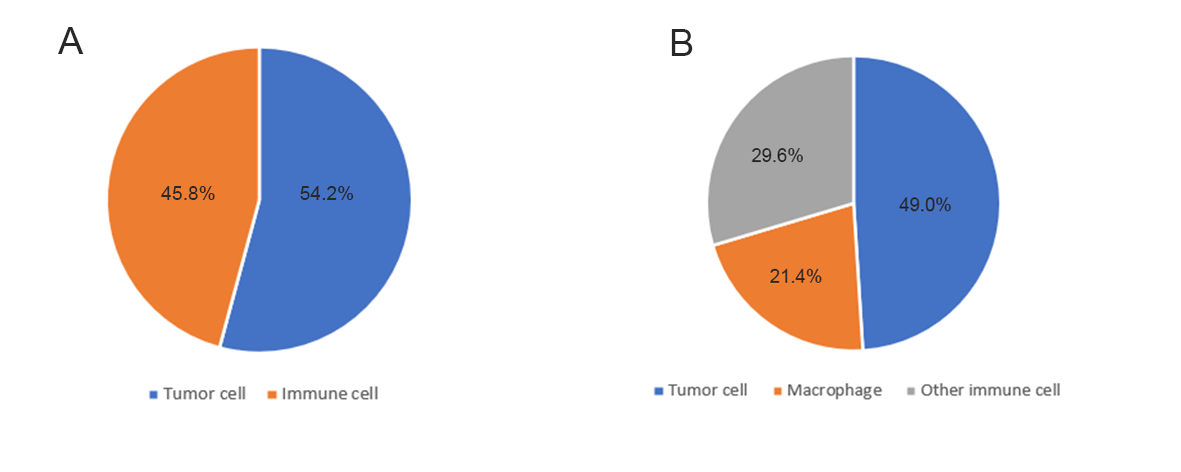
**

**Supplementary Figure S2**. Kaplan–Meier curves of overall survival according to (A) OX40 mRNA expression level and (B) OX40L mRNA expression level in The Cancer Genome Atlas database.


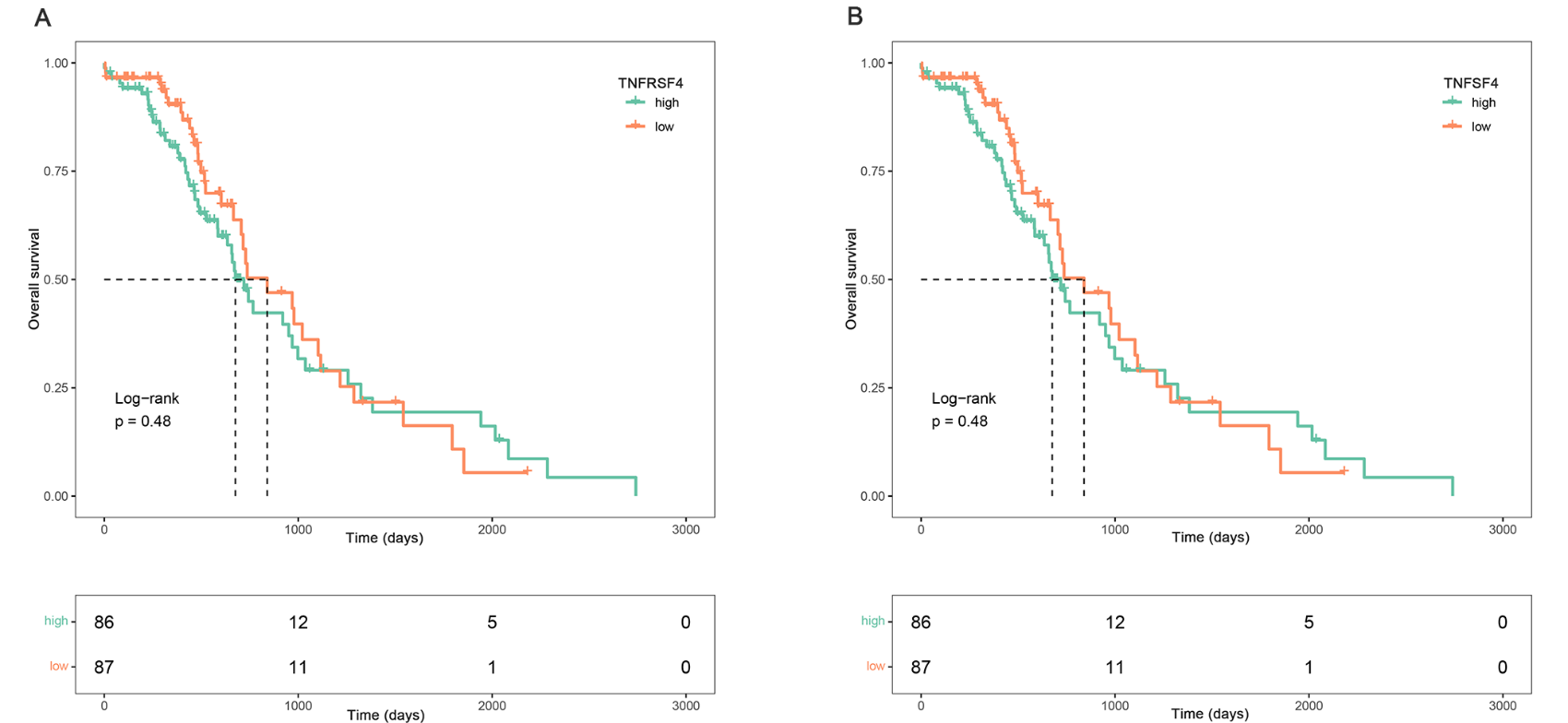


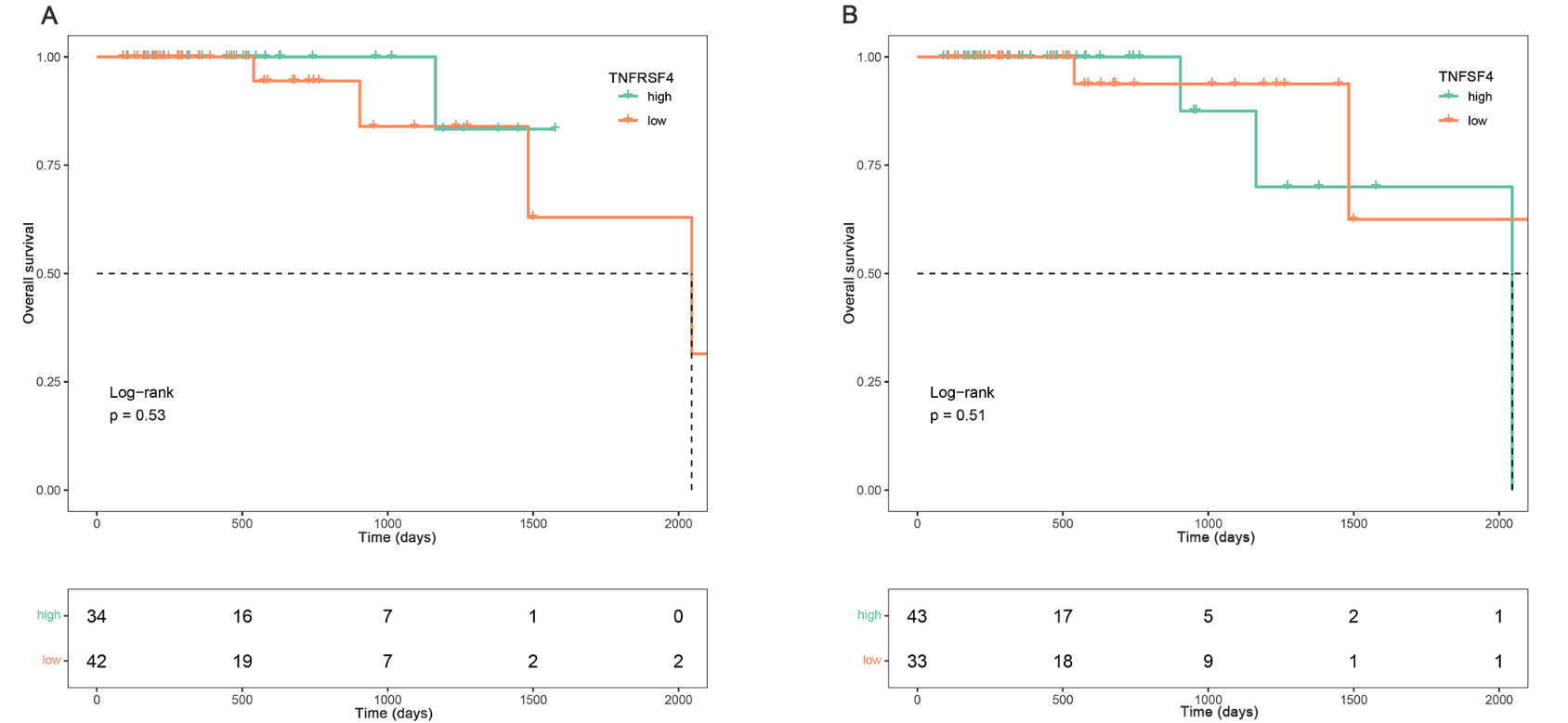
**Supplementary Figure S3.** Kaplan–Meier curves of overall survival according to (A) OX40 mRNA expression level and (B) OX40L mRNA expression level in the International Cancer Genome Consortium database.

**Supplementary Figure S4.** Kaplan–Meier curves according to OX40 and OX40L expression in pT1–pT2 and pT3 groups. OX40+IC was related to a superior disease-specific survival (DSS) (A) and progression-free survival (PFS) (B) compared to OX40-ICs in the pT1–pT2 group. OX40L+ TCs were related to a superior DSS (C) and PFS (D) compared to OX40L− TCs in the pT1-pT2 group. Moreover, OX40L+ macrophages were related to a superior DSS (E) and PFS (F) compared to OX40L− macrophages in the pT1–pT2 group. Corresponding images G–L were observed in the pT3 group


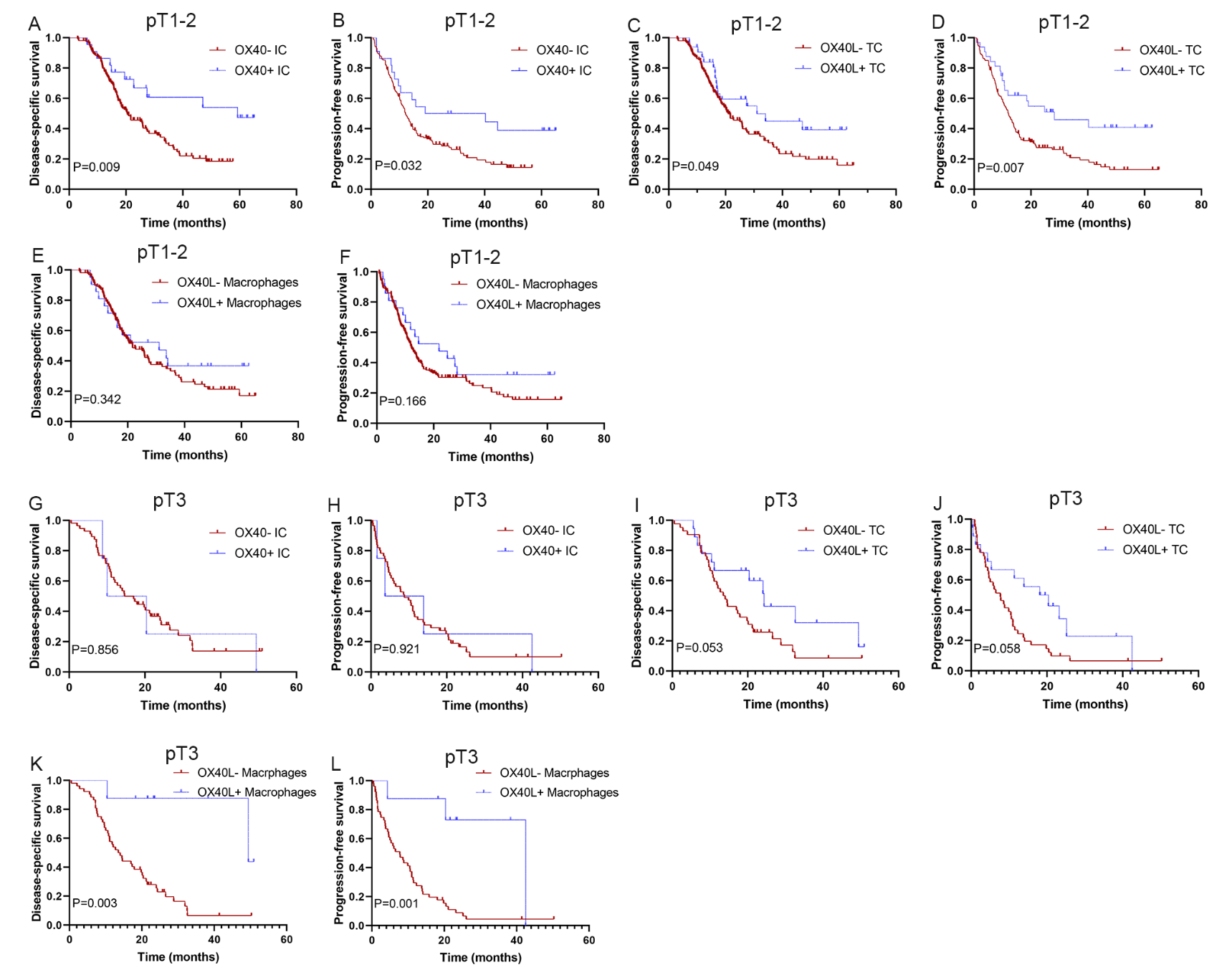


**Supplementary Figure S5.** Kaplan–Meier curves according to OX40 and OX40L expression in programmed death-ligand 1 (PD-L1)-negative and PD-L1-positive groups. OX40+ ICs were related to a better disease-specific survival (DSS) (A) and progression-free survival (PFS) (B) compared to OX40-ICs in the PD-L1-negative group. OX40L+ TCs were related to a better DSS (C) and PFS (D) compared to OX40L− TCs in the PD-L1-negative group. Moreover, OX40L+ macrophages were related to a better DSS (E) and PFS (F) compared to OX40L− macrophages in the PD-L1-negative group. Corresponding images G–L were observed in the PD-L1-positive group.


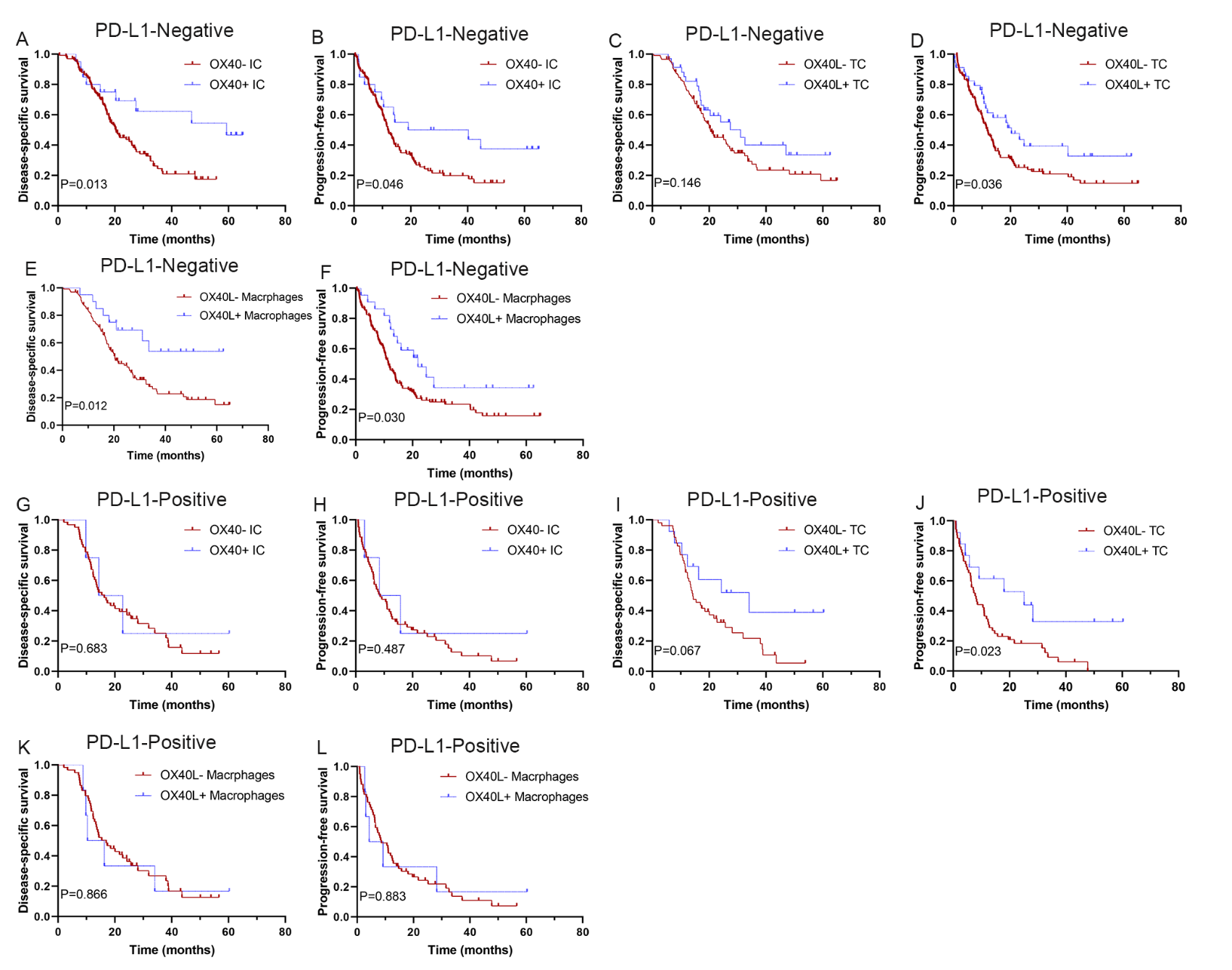

Supplement: Supplementary file 1 [file DataSheet_1.docx]
